# The Impact of Short-Term Video Games on Performance among Children with Developmental Delays: A Randomized Controlled Trial

**DOI:** 10.1371/journal.pone.0149714

**Published:** 2016-03-16

**Authors:** Ru-Lan Hsieh, Wen-Chung Lee, Jui-Hsiang Lin

**Affiliations:** 1 Department of Physical Medicine and Rehabilitation, Shin Kong Wu Ho-Su Memorial Hospital, Taipei, Taiwan; 2 Department of Physical Medicine and Rehabilitation, School of Medicine, College of Medicine, Taipei Medical University, Taipei, Taiwan; 3 Institute of Epidemiology and Preventive Medicine, College of Public Health, National Taiwan University, Taipei, Taiwan; IRCCS E. Medea, ITALY

## Abstract

This prospective, randomized controlled study investigated the effects of short-term interactive video game playing among children with developmental delays participating in traditional rehabilitation treatment at a rehabilitation clinic. One hundred and one boys and 46 girls with a mean age of 5.8 years (range: 3 to 12 years) were enrolled in this study. All patients were confirmed to suffer from developmental delays, and were participating in traditional rehabilitation treatment. Children participated in two periods of 4 weeks each, group A being offered intervention of eight 30-minute sessions of interactive video games in the first period, and group B in the second, in addition to the traditional rehabilitation treatment. The physical, psychosocial, and total health of the children was periodically assessed using the parent-reported Pediatric Quality of Life Inventory-Generic Core Scales (PedsQL); and the children’s upper extremity and physical function, transfer and basic mobility, sports and physical functioning, and global functioning were assessed using the Pediatric Outcomes Data Collection Instrument. Parental impact was evaluated using the PedsQL-Family Impact Module for family function, PedsQL-Health Satisfaction questionnaire for parents’ satisfaction with their children’s care and World Health Organization-Quality of Life-Brief Version for quality of life. Compared with the baseline, significant improvements of physical function were observed in both groups (5.6 ± 19.5, p = 0.013; 4.7 ± 13.8, p = 0.009) during the intervention periods. No significant improvement of psychosocial health, functional performance, or family impact was observed in children with developmental delays. Short-term interactive video game play in conjunction with traditional rehabilitation treatment improved the physical health of children with developmental delays.

***Trial Registration*:** ClinicalTrials.gov NCT02184715

## Introduction

Health-related quality of life (HRQOL) is the individualized perception of physical, mental, social and role functioning related to the personal consequences of various medical conditions and treatment [1]. Children with developmental delays have a significantly lower HRQOL and health status than children with typical development [2]. The parents have higher family impact, including lower HRQOL and family function, and higher psychological distress than the parents of children with typical development [2]. Children with developmental delays who exhibit higher functioning levels exhibit enhanced HRQOL and lower family impact [2]. Early intensive treatments are highly beneficial for children with disabilities [2].Determining how to improve the health and functional performance, along with a reduced family impact, of children with developmental delay is crucial.

Interactive video games have recently become popular because they are enjoyable and low cost [3]. However, they exert both positive and negative effects on children [4]. Excessive play and violent video games are associated with aggressive behavior, hostile effects, attention problems, hyperactivity, somatic complaints, reductions in prosocial behavior, family interaction problems, scholastic problems [5,6], and considerable impairment of sleep [7,8]. Time spent playing is associated with gender, age, parental factors and physical environmental factors, such as having a television in the bedroom of preschool children [9]. Conversely, interactive prosocial video games enable the player to physically interact with images on a screen by engaging in various activities and movements [10]. In addition, such games provide several effective systems to motivate users, such as rewards, interative advances based on consecutive challenges, and options to customize in a personalized way [11]. These games are more fun, safe, entertaining and enjoyable than traditional forms of physical activity [12,13].

Previous studies have reported that interactive-video-game playing improves the motor skills, postural control and upper extremity motor function in children with Down syndrome, degenerative ataxia, or cerebral palsy [14,15,16]. The direct participation of parents in interactive-video-game playing can motivate children to learn new skills [17]. While improvements in single leg stance and grip strength were noted in children with developmental delays by interactive-video-game playing [18], the effects on HRQOL and functional performance among these children, as well as their family impact remain undetermined.

Therefore, a prospective study was conducted to investigate the effects of short-term interactive-video-game playing on children with developmental delays attending traditional rehabilitation treatment. We hypothesized that an additional 4-week phase of interactive-video-game playing with family participation would improve the functioning and HRQOL of these children, and also reduce the impact on their families.

## Materials and Methods

### Participants

This study was conducted at Shin Kong Wu Ho-Su Memorial Hospital, a teaching hospital with 921 beds located in Northern Taiwan, from Jan 2009 to Dec 2009. Developmental delay was diagnosed according to performance scores on age-appropriate, norm-referenced, and standardized tests, with scores falling below two standard deviations (or more) of the mean score for the general population (control children), indicating developmental delay. The assessment tools commonly used for children with developmental delays include the Bayley III Scales of Infant and Toddler Development, Gross Motor Function Measure, Peabody Developmental Motor Scales, Child Expression Evaluation Tool, Preschool Language Evaluation Tool, and Chinese Wechsler Intelligence Scale for Children (Third Edition).

Eligibility criteria for the study were as follows: ages 3 to 12 years; children diagnosed with developmental delays in the domains of gross motor function, fine motor function, speech and language, cognition, or social and emotional function, and attending traditional rehabilitation treatment; and the availability of children to attend with their parents two stages of interactive-video- game playing for 8 weeks. Treating physicians informed eligible families about the study in a clinic for children with developmental delays. If parents were interested in the study, they were free to participate. All parents provided written informed consent for themselves and their children to participate in the study (S1 Text). This study was approved by the Institutional Review Board for the Protection of Human Subjects at Shin Kong Wu Ho-Su Memorial Hospital (IRB reference number: 97E-037) (S2 Text), and the study was performed in accordance with the World Medical Association Declaration of Helsinki. The methods were carried out in accordance with the approved guidelines (S3 Text and S4 Text). This study was registered at ClinicalTrials.gov under the Unique Identifier: NCT02184715 on July 8, 2014 (S5 Text) (Due to no requirement for clinical trial registration of clinical researches in Taiwan, this study was retrospectively registered).

### Protocol and procedures

This study was a prospective, randomized controlled trial. The participants were randomly assigned to either Group A or Group B based on computer-generated random numbers. The principle of block randomization was used to assign patients to the groups, with a block size of ten. Allocation was initially concealed. An envelope was opened for all consecutive participants to reveal their group assignment at the time when they were recruited into the study. One physician enrolled all participants, and the other physician generated the allocation sequence and assigned participants to their groups.

**Fig 1:**
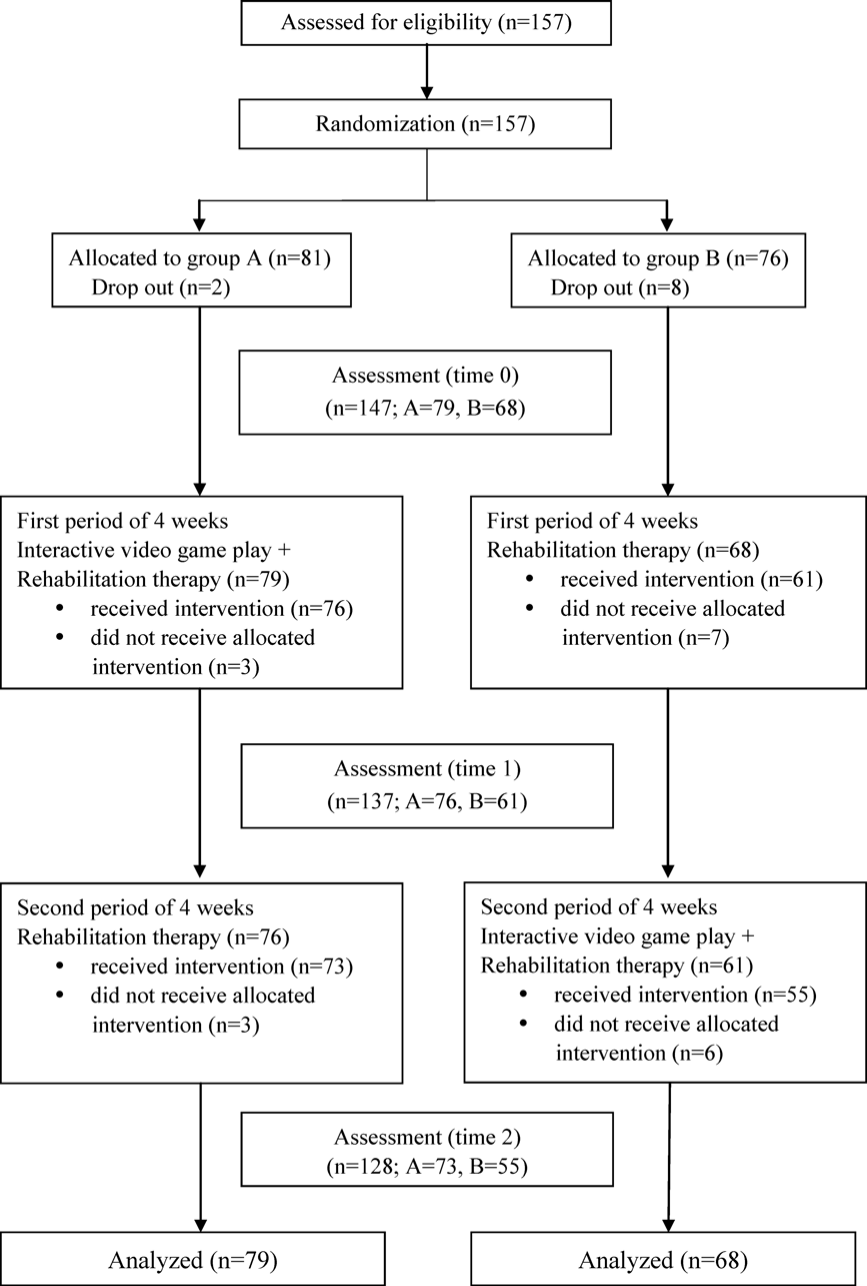
Trial profile.

Group A participants received traditional rehabilitation treatment and the add-on eight 30-minutes sessions of interactive-video-game playing for 4 weeks, followed by traditional rehabilitation treatment alone for 4 weeks. Group B participants received traditional rehabilitation treatment alone for 4 weeks, followed by traditional rehabilitation treatment and the add-on eight 30-minutes sessions of interactive-video-game playing for 4 weeks (Fig 1). This was therefore a crossover study (Group A: the add-on first followed by traditional treatment; Group B: traditional treatment first followed by the add-on). The traditional rehabilitation sessions focused on facilitation of balance, coordination, movement transitions, walking, gross and fine motor control, and sensory-integration. During the add-on intervention periods, the participants continued to attend traditional rehabilitation treatment including physical therapy, occupational therapy, and/or speech therapy.

### Intervention

**Fig 2:**
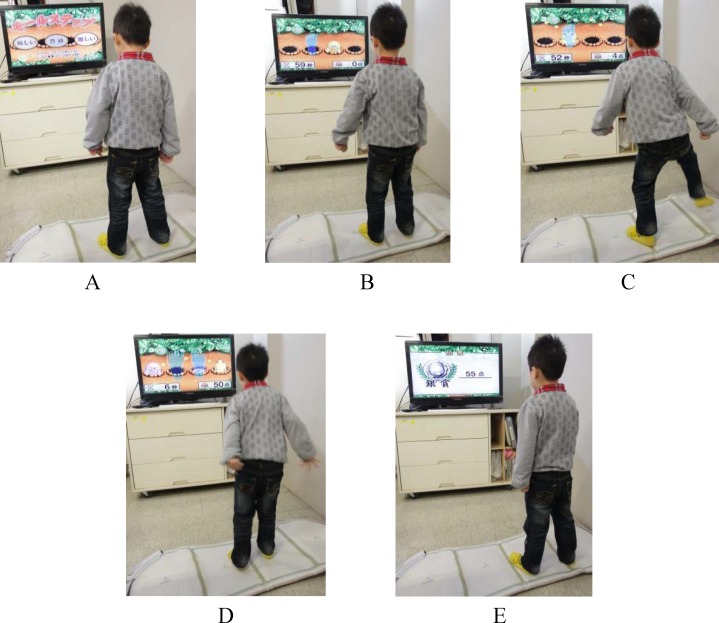
One example of the game playing “whack-a-mole”. In this mole-themed version of “whack-a-mole”, a step-sensing pad is placed on the floor, and the participant treads on the pad as fast as possible when virtual moles randomly appear on the holes of the TV screen. The game has three difficulty levels (A). The higher the difficulty level is, the faster the moles appear, and the more moles appear simultaneously. Level I has one mole (B shows that the player failed to stomp the mole into the hole with his left foot; C shows the player successfully stomping the mole into the hole with his left foot), Level II has two moles, and Level III has three moles (D shows the player successfully stomping a mole with his right foot but failing to stomp another mole with his left foot). The score of successful stomps appears in the right bottom corner of the TV screen with auditory feedback, and the remaining time appears in the left bottom corner of the TV screen during play. After the game has finished, the final score appears on the screen (E).

Each participant engaged in interactive-video-game playing and used of sensors attached to the limbs during the 4-week, hospital-based training period (30 min, two sessions per week, with a total of eight sessions over 4 weeks). The video games were played using a television screen and a sensor by the interactive prosocial commercialized video game system (Hot Plus; Supreme Investment Co., Taipei, Taiwan, ROC). The Hot Plus contained a console which used cartridges and wireless controllers. The controllers were shaped like sports equipment (such as bowling ball, tennis racquets or baseball bats), gloves, or step-sensing pad. Users interacted with the video game according to the representations on the television screen through infrared photo sensing in the controllers. It provided 24 games involving repeated movements and tasks that entailed moving the upper limbs, lower limbs, and trunk [3].The games were classified into the categories of goal-directed entertainment, upper-limb training, lower-limb training and brainstorming in a virtual environment focused on the participant’s balance, walking, strength, weight bearing, reach and grasping, eye-hand coordination, cognition, and attention [3]. The games offered different levels of difficulty by requiring the participants to respond to novel situations and motivating them with aural and visual feedback and rewards (Fig 2). The researcher observed each child’s functional performance, abilities, and interests, and then selected particular games at appropriate levels of difficulty. The selected games were enhanced and maintained by children’ enthusiasm and interest for play as much as possible. A single researcher conducted all the interventions.

### Outcome measures

Following the recruitment and baseline assessment, the outcome measures were assessed before treatment (Time 0), at the end of the first intervention in the fourth week (Time 1) and at the end of the second intervention in the eighth week (Time 2). The investigator conducting the assessment was blinded to the allocation of each participant. Fig 1 provides an overview of the trial flow.

#### Health-related quality of life and functional performance of the children

The Pediatric Quality of Life Inventory Generic Core Scales (PedsQL; parent proxy-report format) was used to measure the pediatric HRQOL according to a parent proxy-report [19,20]. The physical health summary scores, psychosocial health summary scores and total scale scores were calculated. The scores range from 0 to 100, with higher scores representing higher HRQOL. Several Chinese studies have reported satisfactory feasibility, reliability and validity for PedsQL [21,22].

The parent-report form of the Pediatric Outcomes Data Collection Instrument (PODCI) was used to assess the functional performance of the children [23]. This study focused on the four functional performance domains in the PODCI: upper extremity and physical function, transfer and basic mobility, sports and physical functioning, and global functioning. The scores range from 0 to 100; higher scores represent higher levels of functioning. Our previous studies have proven the reliability of the Chinese version of the PODCI [22,24].

#### Family impact

Family impact was evaluated using three instruments. The first instrument was the PedsQL Family Impact Module used to assess family functioning, the Chinese version of which exhibits satisfactory interrater and intrarater reliability [22,25]. The scores range from 0 to 100; higher scores indicate higher functioning.

The second instrument was the PedsQL Health Satisfaction questionnaire used to assess the parents’ satisfaction with their child’s health care, the Chinese version of which exhibits satisfactory reliability [22,25]. The scores range from 0 to 100; higher scores indicate greater satisfaction with health care.

The third instrument was the Chinese version of the World Health Organization Quality of Life Brief Version (WHOQOL-BREF) [26] questionnaire, which was used to evaluate the parents’ QOL and which exhibits high interrater and intrarater

Reliability [22]. The questionnaire comprises physical, psychological and social relationships, and environmental domains [27]. The scores range from 0 to 100; higher scores indicate higher QOL.

### Statistical analysis

Statistical analyses were performed using SAS Version 9.2. The results were expressed as mean ± standard deviation. Chi-squared or *t* tests were used to compare the differences in the data between Groups A and B according to their demographic and baseline variables. Changes in outcome measures of different time points in both groups were conducted by paired *t* tests. Between groups differences in the changes of outcome measures were conducted using Student t test. Repeated measurement analysis of variance (ANOVA) was also conducted to test group effect, time effect and group x time interaction effect, respectively. Estimates of effect size and 95% confidence intervals were reported. The following interpretation for the magnitude of the effect size d is suggested: 0–0.1, no effect; 0.2–0.4, a small effect; 0.5–0.7, an intermediate effect; 0.8 and higher, a large effect. All the statistical tests were two-tailed and α = 0.05. The sample size for the paired *t* tests was calculated assuming a standardized effect (mean difference/standard deviation) of 0.4. Because the β value was 80%, the number of patients in each treatment arm was 51 [28]. Intention to treat analysis (last observation carried forward) was also performed.

## Results

In total, 157 children were assessed for eligibility. Of the 157 potential participants, 8 declined to participate for unstated personal reasons and 2 participants were excluded due to unavailability; 147 participants completed the initial assessment (time 0). In this study, 101 boys and 46 girls, with a mean age of 5.8 years (range: 3–12 years), were enrolled. Groups A and B comprised 79 and 68 participants, respectively. After the initial evaluation, 3 participants in Group A and 7 participants in Group B refused to participate in the first period of intervention due to personal time reasons. Three participants from Group A and 6 from Group B were lost to follow-up after the second period of intervention due to personal time reasons or unspecified reasons. In Group A, 76 completed the first period of assessment (Time 1), and 73 participants completed the second period of assessment (Time 2). In Group B, 61 participants completed the first period of assessment (Time 1), and 55 participants completed the second period of assessment (Time 2). The dropout rate was 12.9%. No adverse event was reported during the intervention.

No statistically significant differences were observed in the demographic data between the groups (Table 1). Except for the higher social relationship scores observed in Group A (59.8 ± 15.0 vs. 54.5 ± 13.9, p = 0.027), no significant differences were observed in the baseline scores of the PedsQL, PODCI, PedsQL-Family Impact Module, PedsQL-Health Satisfaction, and in the physical, psychological and environmental domains of the WHOQOL-BREF between the groups (Table 2).

**Table 1:** Basic demographics of the participating children and their parents.

Variables	Group A (n = 79)	Group B (n = 68)	P-value
Children			
Gender			
Male	29 (37%)	17 (25%)	0.127
Female	50 (63%)	51 (75%)	
Age	6.1 ± 2.3	5.6 ± 1.8	0.104
Diagnosis			
ADHD	14 (20%)	17 (26%)	0.738
Autism spectrum disorder	5 (7%)	7 (10%)	
Cerebral palsy	10 (14%)	11 (17%)	
Mental retardation	11 (16%)	9 (14%)	
Unclassified	30 (43%)	22 (33%)	
Delayed developmental domains			
Global	45 (67%)	49 (77%)	0.534
Cognition	24 (36%)	21 (33%)	0.235
Speech–language	27 (40%)	30 (47%)	0.491
Gross motor	37 (55%)	29 (45%)	0.525
Fine motor	27 (40%)	29 (45%)	0.581
Emotion	7 (11%)	7 (11%)	0.382
Social adjustment	9 (14%)	5 (8%)	0.281
Personal interaction	8 (12%)	5 (8%)	0.413
Sensory integration	14 (21%)	16 (25%)	0.848
Parents			
Education			
< Ninth grade	7 (9%)	6 (9%)	0.984
≥ Ninth grade	72 (91%)	61 (91%)	
Employed			
Yes	31 (49%)	19 (32%)	0.056
Married			
Yes	73 (92%)	60 (88%)	0.391
Comorbidity			
Yes	14 (20%)	12 (20%)	1.000
Smoking			
Yes	12 (15%)	7 (10%)	0.378

Data are expressed as number (percentage) or mean ± standard deviation. ADHD, attention deficit hyperactivity disorder.

**Table 2:** Scores for the participating children and their parents before treatment.

Variables	Group A (n = 79)	Group B (n = 68)	P-value
Children			
PedsQL			
Physical	67.0 ± 25.6	65.2 ± 25.7	0.666
Psychosocial	63.1 ± 20.4	62.7 ± 19.2	0.901
Total	64.2 ± 21.1	63.9 ± 19.7	0.933
PODCI			
Upper extremity and physical function	82.0 ± 21.3	74.7 ± 25.8	0.063
Transfer and basic mobility	89.9 ± 22.7	86.0 ± 25.7	0.336
Sports and physical function	79.1 ± 23.5	76.1 ± 27.4	0.387
Global function	83.9 ± 19.0	78.6 ± 22.7	0.132
Parents			
PedsQL-Family Impact Module	61.7 ± 16.4	59.4 ± 17.8	0.428
PedsQL-Health Satisfaction	76.9 ± 18.9	72.7 ± 18.4	0.178
WHOQOL-BREF			
Physical	62.3 ± 13.2	60.1 ± 14.2	0.331
Psychological	53.5 ± 14.9	49.9 ± 21.9	0.255
Social relationships	59.8 ± 15.0	54.4 ± 13.9	0.027*
Environment	55.9 ± 13.4	52.9 ± 14.0	0.192

Data are expressed as mean ± standard deviation.

PedsQL, Pediatric Quality of Life Inventory-Generic Core Scale (parent proxy-report format); PODCI, Pediatric Outcomes Data Collection Instrument; PedsQL-Family Impact Module, Pediatric Quality of Life Inventory Family Impact Module; PedsQL-Health Satisfaction, Pediatric Quality of Life Inventory Health Satisfaction; WHOQOL-BREF, World Health Organization Quality of Life Brief Version.

* P < 0.05

**Fig 3:**
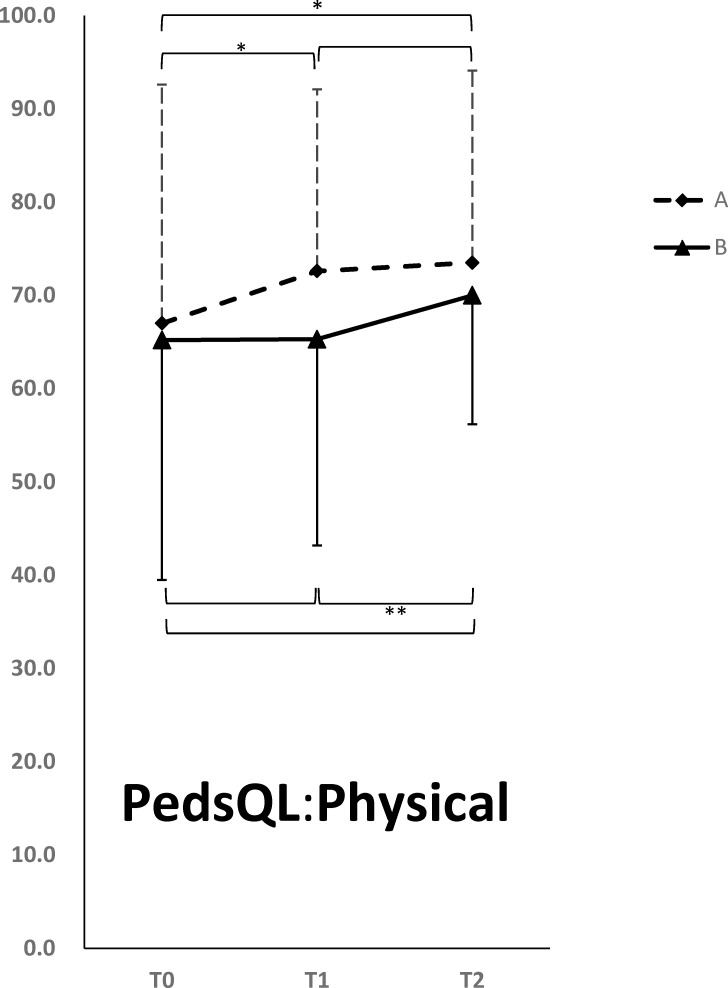
The changes of PedsQL physical health summary scores associated with video game playing. Solid square, Group A; solid triangle, Group B. Abbreviations: PedsQL, Pediatric Quality of Life Inventory-Generic Core Scales; t_0_, before treatment; t_1_, at the end of the first intervention during the fourth week; t_2_, at the end of the second intervention during the eighth week. *p < 0.05; **p < 0.01.

Tables 3 and 4 summarize the changes in outcome measures of different time points in both groups and between groups differences in the changes of outcome measures in participating children and their parents, respectively. Since there were three time points, and we compared the results at Time 1 and Time 0; Time 2 and Time 1; and Time 2 and Time 0, we used Bonferroni’s correction to account for multiple testing (a total of three tests, therefore the α level was adjusted to 0.05/3 = 0.0167). Compared with the baseline assessment results, Group A participants exhibited significant improvement in physical health as measured by PedsQL at Time 1 (5.6 ± 19.5, p = 0.013) (Fig 3). The significant improvement of physical health persisted to Time 2 (5.6 ±18.4, p = 0.011). The improvement of physical health in Group A has small effect sizes of 0.30 (to time 1) and 0.32 (to time 2). Although psychosocial health (4.4 ±13.8, p = 0.013) and total score (4.6 ±13.9, p = 0.01) from baseline as measured by PedsQL to Time 2 were also significant after Bonferroni’s correction, the magnitudes of the effect size d were less than 0.2. (Table 3). Compared with pre-intervention, Group B participants exhibited significant improvement in their physical health (4.7 ± 13.8, p = 0.009) (Fig 3) at Time 2 but not at Time 1 (Table 3). The results of repeated measurement ANOVA were consistent with previous results, where we found a significant time effect (p = 0.015), but no group effect (p = 0.128), and group x time interaction effect (p = 0.257). No statistically significant changes were observed in family impact for participating parents after the intervention, including family function as measured by PedsQL Family Impact Module, parents’ satisfaction with their child’s health care as measured by PedsQL Health Satisfaction, and parents’ QOL as measured by WHOQOL-BREF (Table 4).

**Table 3:** Changes in the scores for the participating children after intervention.

Variables	Changes after intervention								
	Time _1–0_			Time _2–1_			Time _2–0_		
	MD(95% CI)	Cohen's d	P	MD(95% CI)	Cohen's d	P	MD(95% CI)	Cohen's d	P
PedsQL									
Physical									
A	5.6(-0.52, 0.11)	0.30^a^	0.013*	0.9(-0.12, 0.50)	0.05	0.717	5.6(-0.31, 0.30)	0.32^a^	0.011*
B	0.1(-0.34, 0.32)	0.005	0.966	4.7(-0.48, 0.18)	0.25^a^	0.009*	5.0(-0.50, 0.16)	0.23^a^	0.101
A vs B	1.8(-0.25, 0.39)	0.07	0.666	6.8(-0.07, 0.57)	0.13	0.146	0.3(-0.17, 0.47)	0.08	0.616
Psychosocial									
A	3.4(-0.48, 0.13)	0.17	0.036	0.8(-0.29, 0.32)	0.15	0.685	4.4(-0.47, 0.15)	0.15	0.013*
B	1.3(-0.44, 0.23)	0.11	0.527	2.1(-0.39, 0.27)	0.06	0.337	3.2(-0.52, 0.15)	0.18	0.109
A vs B	0.4(-0.30, 0.34)	0.03	0.901	1.7(-0.25, 0.40)	0.08	0.640	0.2(-0.31, 0.33)	0.01	0.951
Total									
A	4.2(-0.52, 0.11)	0.21^a^	0.017	0.6(-0.28, 0.33)	0.17	0.777	4.6(-0.48, 0.14)	-0.03	0.010*
B	0.6(-0.38, 0.28)	0.05	0.805	3.2(-0.44, 0.22)	0.20^a^	0.102	3.9(-0.51, 0.15)	0.11	0.084
A vs B	0.3(-0.31, 0.33)	0.01	0.933	3.5(-0.17, 0.47)	0.15	0.382	0.4(-0.31, 0.34)	0.01	0.933
PODCI									
Upper extremity and physical function									
A	1.6(-0.39, 0.22)	0.09	0.163	0.8(-0.33, 0.29)	0.02	0.415	2.4(-0.41, 0.20)	0.11	0.008*
B	-1.0(-0.34, 0.32)	0.01	0.614	2.5(-0.40, 0.26)	0.08	0.149	1.1(-0.42, 0.25)	0.09	0.659
A vs B	7.3(-0.02, 0.63)	0.31^a^	0.063	8.8(0.06, 0.71)	0.38^a^	0.022	7.4(-0.002, 0.65)	0.32^a^	0.052
Transfer and basic mobility									
A	1.2(-0.37, 0.25)	0.06	0.039	-0.4(-0.37, 0.25)	-0.03	0.393	1.0(-0.33, 0.28)	0.03	0.154
B	-1.9(-0.38, 0.28)	-0.04	0.400	1.4(-0.33, 0.33)	0.003	0.676	-0.7(-0.28, 0.38)	-0.05	0.735
A vs B	3.9(-0.16, 0.48)	0.16	0.336	6.8(-0.09, 0.55)	0.22^a^	0.181	6.0(-0.1, 0.54)	0.21^a^	0.204
Sports and physical function									
A	-0.6(-0.29, 0.32)	-0.01	0.630	0.3(-0.31, 0.31)	0	0.821	-0.1(-0.29, 0.32)	-0.01	0.967
B	-0.1(-0.35, 0.31)	0.02	0.937	2.3(-0.36, 0.30)	0.03	0.153	2.2(-0.38, 0.29)	0.05	0.165
A vs B	3.0(-0.20, 0.44)	0.14	0.387	2.2(-0.23, 0.41)	0.09	0.583	1.5(-0.26, 0.38)	0.05	0.751
Global function									
A	-0.03(-0.35, 0.26)	0.05	0.968	0.03(-0.26, 0.35)	-0.03	0.971	0.2(-0.31, 0.31)	0	0.814
B	-0.8(-0.42, 0.24)	0.01	0.552	2.6(-0.37, 0.30)	0.08	0.063	1.4(-0.47, 0.20)	0.09	0.323
A vs B	5.3(-0.07, 0.58)	0.25^a^	0.132	5.9(-0.02, 0.62)	0.29^a^	0.086	3.5(-0.15, 0.49)	0.16	0.325

Data are expressed as MD (95% CI) = mean difference (95% confidence level). PedsQL, Pediatric Quality of Life Inventory-Generic Core Scale (parent proxy-report format); PODCI, Pediatric Outcomes Data Collection Instrument.

^a^ Cohen's d ≥ 0.2

* P < 0.0167 (after Bonferroni’s correction).

**Table 4:** Changes in the scores for the participating parents after intervention.

Variables	Changes after intervention								
	Time _1–0_			Time _2–1_			Time _2–0_		
	MD(95% CI)	Cohen's d	P	MD(95% CI)	Cohen's d	P	MD(95% CI)	Cohen's d	P
PedsQL Family Impact Module									
A	0.7(-0.33, 0.28)	0.03	0.557	0.4(-0.37, 0.25)	0.06	0.753	1.1(-0.39, 0.22)	0.09	0.433
B	0.2(-0.34, 0.32)	0.02	0.895	3.4(-0.55, 0.12)	0.22^a^	0.037	3.9(-0.56, 0.10)	0.23^a^	0.035
A vs B	2.3(-0.18, 0.45)	0.13	0.428	2.5(-0.18, 0.46)	0.14	0.408	-0.4(-0.34, 0.30)	0.02	0.900
PedsQL Health Satisfaction									
A	-1.1(-0.24, 0.38)	-0.07	0.495	1.7(-0.39, 0.23)	0.08	0.465	-0.8(-0.32, 0.30)	0.01	0.732
B	4.8(-0.57, 0.10)	0.24^a^	0.031	-0.7(-0.27, 0.39)	0.06	0.719	2.2(-0.51, 0.16)	0.18	0.354
A vs B	4.2(-0.10, 0.55)	0.22^a^	0.178	-1.8(-0.41, 0.23)	0.09	0.580	1.0(-0.27, 0.37)	0.05	0.770
WHOQOL-BREF									
Physical									
A	-1.2(-0.19, 0.43)	-0.12	0.331	-1.5(-0.20, 0.42)	-0.11	0.29	-1.5(-0.08, 0.54)	0.23^a^	0.260
B	0.9(-0.41, 0.25)	0.08	0.485	-0.3(-0.35, 0.31)	0.02	0.817	-0.3(-0.43, 0.24)	0.10	0.817
A vs B	2.2(-0.16, 0.48)	0.16	0.331	-0.5(-0.36, 0.28)	0.04	0.823	-2.3(-0.48, 0.16)	0.15	0.369
Psychological									
A	-2.2(-0.17, 0.45)	-0.14	0.099	-0.6(-0.24, 0.37)	-0.06	0.674	-0.6(-0.10, 0.52)	-0.22	0.674
B	0.4(-0.30, 0.37)	0.04	0.869	-1.8(-0.41, 0.25)	-0.08	0.224	-1.8(-0.37, 0.30)	-0.03	0.224
A vs B	3.6(-0.13, 0.51)	0.20^a^	0.255	0.7(-0.28, 0.36)	0.04	0.820	1.1(-0.25, 0.39)	0.06	0.711
Social relationships									
A	-0.6(-0.24, 0.37)	-0.06	0.706	-1.5(-0.24, 0.37)	-0.12	0.229	-1.5(-0.11, 0.50)	-0.20	0.229
B	3.1(-0.56, 0.11)	0.23^a^	0.063	-0.6(-0.32, 0.34)	-0.01	0.675	-0.6(-0.54, 0.12)	0.21^a^	0.675
A vs B	5.4(0.04, 0.69)	0.37^a^	0.027	1.2(-0.24, 0.40)	0.08	0.624	-0.5(-0.35, 0.29)	0.03	0.845
Environment									
A	-1.4(-0.17, 0.45)	-0.13	0.194	-1.6(-0.21, 0.41)	-0.12	0.141	-1.6(-0.07, 0.54)	-0.20	0.141
B	1.3(-0.45, 0.22)	0.12	0.368	-0.8(-0.34, 0.32)	-0.09	0.549	-0.8(-0.45, 0.21)	0.21^a^	0.549
A vs B	3.0(-0.10, 0.54)	0.22^a^	0.192	-0.5(-0.36, 0.28)	0.03	0.848	-2.0(-0.46, 0.18)	0.13	0.429

Data are expressed as MD (95% CI) = mean difference (95% confidence level). PedsQL-Family Impact Module, Pediatric Quality of Life Inventory Family Impact Module; PedsQL-Health Satisfaction, Pediatric Quality of Life Inventory Health Satisfaction; WHOQOL-BREF, World Health Organization Quality of Life Brief Version.

^a^ Cohen's d ≥ 0.2.

The only significant improvement during the intervention periods in both groups was physical health of children and the effect persisted to the end of the 8-week period (Fig 3 and Table 3). We specifically compared the change of scores between Time 1 and Time 0 in Group A (5.6 ± 19.5) and the change of score between Time 2 and Time 1 in Group B (4.7 ± 13.8), and found no difference (p = 0.138), indicating that effects on the add-on treatment in the two groups were the same.

## Discussion

We investigated the additional effects of short-term interactive-video-game playing on the health, functional performance and family impact of children with developmental delays attending traditional rehabilitation treatment. For ethical reasons, we did not include a true control group in the present study. The children underwent the intervention during their traditional rehabilitation treatment. The study results indicated physical health improvement in the children following the 4-week, short-term interactive-video-game playing, and the effect persisted for 4 weeks. However, no considerable alternations were observed in the functional performance or parental impact among these children.

Short-term interactive-video-game playing in conjunction with standard rehabilitation improves the physical health of children with developmental delays. The games were selected by clinical staff in accordance with a therapy tailored on participants’ functional limitations, abilities, interest and needs. Because interactive gaming is interesting, exciting and enjoyable for children, they like to initiate and sustain video-game playing [29,30]. Positron emission tomography scans have proven that endogenous dopamine is released in the human striatum during interactive-video-game playing [31]. Dopaminergic neurotransmission is involved in learning, attention, sensory motor integration and behavior reinforcement through environmental actions or the prediction of rewards or adverse outcomes based on specific stimuli [32]. Based on the concept of neural plasticity, intense practice results in skill acquisition because it facilitates continual reorganization of the nervous system through experience, practice and interactions with various environmental stimuli through auditory and visual biofeedback [14,33]. A previous study indicated that interactive video games support learning in school [11]. Many factors, such as duration and frequency of interactive video games play, contents of video games, type of video game used (e.g., traditional, interactive with sensors, or interactive without sensors), location at which the intervention is conducted (e.g., hospital, community, or home-based), parent participation or not, and the severity of children’s disabilities, etc., might affect the study results. Additional long-term studies that investigate the learning effects of interactive-video-game playing on children with developmental delays with different duration, frequency and video-game contents should be conducted in the future.

A dose effect exists when using virtual reality for rehabilitation in patients with stroke, namely less than 15 hours of treatment were demonstrated to be ineffective [34]. However, recent evidence on video game interventions in children and stroke patients suggests that they have a limited lifespan, as participants get bored and stop using them [35,36]. Therefore, we designed a short-term interactive-video-game playing for children with developmental delays in the present study. The duration and frequency of the intervention would affect the study results. Studies based on at least 12 weeks or longer to assess whether benefits continued beyond 1 month, and whether participants continued to be interested in participating in such an intervention, should be conducted in the future. In addition, a long-term follow-up study (such as at least 1 month or 6 months post-intervention) would have been appropriate to assess whether improvements were sustained after the intervention finished.

High-intensity, repeated task specific interactive game training could improve function and result in better outcome in community frail elderly [37]. The eight sessions conducted at the hospital over the 4-week period of interactive video-game playing revealed no significant improvement in the functional performance or family impact of the children with developmental delays in the present study. The interventions involving short periods of infrequent interactive-video-game playing might have partially contributed to the results. Reportedly, community-based interactive video games exhibit fair acceptability, accessibility and feasibility [38]. Although the long-term adherence to interactive- video-game playing remains uncertain [39], it might provide an opportunity to promote the physical health of children with developmental delays if it can subsequently be translated into parental supervised home therapy programs [39,40]. Lack of interest and motivation of participants in therapeutic treatment of activities and functional performance are identified barriers to rehabilitation [41]. Interactive-video-game playing allowed the participants to engage in the movement and task activities with natural restraints [41]. Further extensive research is warranted to evaluate the economic health care effects of interactive-video-game playing when implementing home-based intervention.

A previous study reported a decline in verbal memory performance after playing computer games, compared with baseline conditions [5]. Based on modern neuroscientific theory, recently acquired knowledge is highly sensitive in the subsequent consolidation period [42]. Strong emotional experiences, such as those elicited by computer games, sustained for hours after learning, might considerably influence memory consolidation [42]. The present study comprised daytime interactive- prosocial-video-game playing that provided goal-directed entertainment without any violent content. The participating children experienced no discomfort or adverse effects (aggravation of aggression, hyperactivity, or sleep problems) during or after the intervention. Therefore, the study results support the recommendation that the content as well as the amount of media exposure should be guided by the rehabilitation and pediatric specialists, school teachers and parents of children with developmental delays [43].

### Limitations

This study has several limitations. First, the diagnoses and developmental disorders of the participants varied. Moreover, the feasibility of extending these results to children with developmental delays with specific diagnosis could not be determined. Furthermore, the inclusion of children with various etiologies for developmental delay was debated in this study because these patients were recruited from clinics for children with developmental delays, thereby representing those assessed and treated in clinical settings. Second, the parent-reported questionnaires were used to assess the health status and functional performance of these children. Because the parents were informed regarding the additional therapy administered to their children, they might have perceived beneficial results of the therapy. Therefore, the parental bias cannot be ruled out. Third, there was a lack of any quantitative and objective measures before and after the intervention. In addition, we did not evaluate the correlation between measures representing specific functions (for instance, upper limb functions) and the training intensity of specific games in the present study. Further research into the effectiveness of interactive video-game-playing for children with developmental delays should be conducted via objective outcome measures, and to identify the correlation between measures representing specific functions and the training intensity of specific games. Lastly, although the therapy was beneficial, additional therapies might exhibit more satisfactory results. Thus, a control with more intensive therapy should be performed in the future, such as a comparison of traditional therapy and additional traditional therapy two times per week versus traditional therapy and additional interactive-video-game therapy two times per week.

### Conclusion

Four weeks of short-term interactive-video-game playing in addition to conventional rehabilitation programs improved the physical health of children with developmental delays. However, no considerable alterations were observed in the functional performance and family impact of the children. Additional long-term effects of interactive-video-game playing on children with developmental delays should be evaluated in future studies.

## Supporting Information

S1 Table Consort list. (PDF)

S1 Text Subject consent form. (DOC)

S2 Text IRB approval statement. (PDF)

S3 Text Clinical trial protocol (original language). (DOC)

S4 Text Clinical trial protocol (main points in English). (DOC)

S5 Text Clinical trial registration. (PDF)
