# Supplementary material for: The Impact of Short-Term Video Games on Performance among Children with Developmental Delays: A Randomized Controlled Trial
Source: PLoS One. 2016 Mar 16;11(3):e0149714. doi: 10.1371/journal.pone.0149714 (PMC4794225; doi:10.1371/journal.pone.0149714)
Supplement: S1 Text — (DOC) [file pone.0149714.s002.doc]

**財團法人新光吳火獅紀念醫院**

**臨床試驗受試者說明及同意書**

(本書表應由計劃主持人親自向受試者說明詳細內容，並請受試者經過慎重考慮後方得簽名)

**□藥品 □醫療器材 ■醫療技術 □其他**

您被邀請參與此研究。本表格提供您有關本研究之相關資訊。研究主持人或其他協同主持醫師將會為您說明研究內容並回答您的任何疑問。

| 計畫名稱：  中文：健康復健互動軟體對發展遲緩兒童復健加成療效研究  英文：**The Additional Therapeutic Effects of Virtual Reality System (Hot Plus) in Children with Developmental Delays** | | | | | | | | | |
| --- | --- | --- | --- | --- | --- | --- | --- | --- | --- |
| 執行單位：新光吳火獅紀念醫院 委託單位/藥廠： | | | | | | | | | |
| 主要主持人： | | | 謝如蘭 | | | | 職稱： | | 復健科主任 |
| 聯絡電話： | | | (02)28332211轉2538 | | | | | | |
| 協同主持人： | | | 李立榕 | | | | 職稱： | | 臨床研究員 |
| 聯絡電話： | | (02)28332211轉450913 | | | | | | | |
| 二十四小時緊急聯絡人電話：李立榕(02)28332211轉450913 | | | | | | | | | |
| **受試者姓名：** | | |  | | | | | | |
| 性別： |  | | | | | 出生日期： | |  | |
| 病歷號碼： | | |  | | | | | | |
| 聯絡通訊地址： | | |  | | | | | | |
| 聯絡電話： | | |  | | | | | | |
| **法定代理人/有同意權人姓名：** | | | | |  | | | | |
| 與受試者關係： | | | |  | | | | | |
| 性別： |  | | | | | 出生日期： | |  | |
| 身份證字號： | | |  | | | | | | |
| 通訊地址： | | |  | | | | | | |
| 聯絡電話： | | |  | | | | | | |

| 1.(藥品／醫療器材／醫療技術)全球(上市／使用)現況簡介：  Hot Plus健康服務－復健互動軟體是由信東生生技其下紹善與日本新世代式會社合作引進，主要是透過電腦銀幕及感應裝置，藉由娛樂遊戲的活動進行身體功能的體能訓練，其中所訓練的部份包含四大項，分別為上肢功能訓練，下肢功能訓練，認知訓練及娛樂活動，可以經由感應裝置包括手套、腳踏墊、感應棒及保球齡球的設計，針對不同程度的發展遲緩兒童提供簡單、普通與難度的活動內容，並可紀錄其分數，提高兒童其從事復健活動的興趣。 | | | | | | | |
| --- | --- | --- | --- | --- | --- | --- | --- |
| 2.試驗目的：  評估使用健康復健互動軟體在發展遲緩兒童之應用成效。 | | | | | | | |
| 3.試驗之主要納入與排除條件：  主要納入條件：  收集本院門診確診為發展遲緩兒童並有穩定接受復健治療者，並記錄所有受試者之資料，包括性別、年齡、發病時間，其他疾病、危險因子評估等相關基本資料，而患童的認知功能必需可以理解指令及操作此互動軟體。  受試者排除條款：  1.年紀小於3歲或大於12歲者  2.其認知功能受損到無法操作最初級的遊戲指令者  3.病人無法配合復健治療者。  4.病人或家屬不願簽署同意書者 | | | | | | | |
| **4.試驗方法及相關檢驗：**  在復健科醫師指導下依患童能力進行「Hot Plus 健康服務-復健互動遊戲」虛擬實境訓練，採取cross-over randomized method的治療，由復健科醫師進行在原有的復健治療方式加上每週兩次，每次三十分鐘的健康復健互動軟體，共為期四週，並於治療前、治療後第四週進行Peds QL, Family impact module, WHO-QOL, CHF-PF28及小兒健問卷等評估。 | | | | | | | |
| **5.可能產生之副作用、發生率及處理方法：**  無 | | | | | | | |
| **6.其他替代療法及說明：**  傳統的復健治療，包括物理、職能及語言治療。 | | | | | | | |
| 7.試驗預期效益：  Hot Plus復健健互動軟體為一種歸類於遊戲復健活動，其使用四大類包含上肢功能訓練，下肢功能訓練、認知功能訓練，及娛樂項目等二十四項遊戲軟體，以類似虛擬實境的遊戲方式，提供發展遲緩兒童一個藉由遊戲而習得經驗、達到治療目的的機會，並明瞭其短期及長期之療效。 | | | | | | | |
| 8.試驗進行中受試者之禁忌、限制與應配合之事項：   - 禁忌：無 - 應配合之事項：受試者均需定期接受療程並回門診接受評估。 | | | | | | | |
| 9.機密性：  本研究將在法律範圍內對您的檢查結果及疾病診斷將妥善保密。您的姓名將以一個研究號碼取代。除了有關機構依法調查、監測者、稽核者與本院醫學倫理暨人體試驗委員有權檢閱原始醫療紀錄外，我們會小心維護您的隱私。試驗結果即使發表，您的身分仍將受到保密。 | | | | | | | |
| 10.損害賠償與保險：   - 如依本研究所訂臨床試驗計畫，因而發生不良反應或傷害，由 新光醫院___依法負損害賠償責任。但本受試者同意書上所記載之不良反應，或這些不良反應所造成之預期傷害，將不予賠償或補償。 - 除法定賠償及醫療照顧外，本研究不提供其他形式之賠償或補償。若您不願意接受這樣的風險，請勿參加試驗。 - 如依本研究所訂臨床試驗計劃，因而發生不良反應或傷害，本醫院願意提供專業醫療照顧及醫療諮詢。您不必負擔治療不良反應或傷害之必要醫療費用。 - 您不會因為簽署本同意書，而喪失在法律上的任何權利。 - □本研究有投保責任保險　■本研究未投保責任保險 | | | | | | | |
| 11.受試者權利：   1. 試驗過程中，與您的健康或是疾病有關，可能影響您繼續接受臨床試驗意願的任何重大發現，都將即時提供給您。 2. 如果您在試驗過程中對試驗工作性質產生疑問，對身為患者之權利有意見或懷疑因參與研究而受害時，可與本院之醫學倫理暨人體試驗委員會聯絡請求諮詢，其電話號碼為：0968996027。 3. 為進行試驗工作，您必須接受 謝如蘭 醫師的照顧。如果您現在或於試驗期間有任何問題或狀況，請不必客氣，可與本院 復健 科的 謝如蘭 醫師聯絡，電話為0968996027。 4. 本同意書一式 2 份，醫師已將同意書副本交給您，並已完整說明本研究之性質與目的。 謝如蘭 醫師已回答您有關藥品與研究的問題。 | | | | | | | |
| 12.試驗之退出與中止：  您可自由決定是否參加本試驗；試驗過程中也可隨時撤銷同意，退出試驗，不需任何理由，且不會引起任何不愉快或影響其日後醫師對您的醫療照顧。試驗主持人或贊助廠商亦可能於必要時中止該試驗之進行。 | | | | | | | |
| 13.簽章   1. 主要主持人、協同主持人或代理主持人已詳細解釋有關本研究計畫中上述研究方法的性質與目的，及可能產生的危險與利益。   主要主持人/協同主持人/代理主持人簽章：  日期：□□□□年□□月□□日   1. 受試者已詳細瞭解上述研究方法及其所可能產生的危險與利益，有關本試驗計畫的疑問，業經計畫主持人詳細予以解釋。本人同意接受為臨床試驗計畫的自願受試者。   受試者簽章：  法定代理人簽章：  有同意權人簽名：  日期：□□□□年□□月□□日   1. 如您不是受試者或其法定代理人，但因事實需要，受試者或其法定代理人(暫時)無法簽署本同意書而需由您代簽。請用正楷書寫您的姓名，並指出您與受試者的關係： | | | | | | | |
|  | (1) | 姓名： |  | | | | |
|  |  | 關係： | | | | | |
|  |  | 身份證字號： | □□□□□□□□□□ | 聯絡電話： | | □□□□□□□□□□ | |
|  |  | 通訊地址： |  | | | | |
|  |  | 簽章： |  | 日期： | □□□□年□□月□□日 | | |
|  | (2) | 見證人(非本人或法定代理人簽章，則須另具見証人一名)： | | | | |  |
|  |  | 姓名： |  | | | | |
|  |  | 身份證字號： | □□□□□□□□□□ | 聯絡電話： | | □□□□□□□□□□ | |
|  |  | 通訊地址： |  | | | | |
|  |  | 簽章： |  | 日期： | □□□□年□□月□□日 | | |
