# Supplementary material for: The Impact of Short-Term Video Games on Performance among Children with Developmental Delays: A Randomized Controlled Trial
Source: PLoS One. 2016 Mar 16;11(3):e0149714. doi: 10.1371/journal.pone.0149714 (PMC4794225; doi:10.1371/journal.pone.0149714)
Supplement: S2 Text — (PDF) [file pone.0149714.s003.pdf]

財團法人
新光吳火獅紀念醫院
SHIN KONG WU HO-SU MEMORIAL HOSPITAL

醫學倫理暨人體試驗審查委員會

同意臨床試驗證明書

試驗計畫名稱：健康復健互動軟體對發展遲緩兒童復健加成療效研究
計畫書版本/日期：第一版/971027
計畫書編號/本會審查編號：97E-037
試驗總主持人：　謝如蘭醫師
受試者同意書版本/日期：第一版/971027
主持人手冊/日期：NA
通過會期：980115
試驗有效期限：2009 年 1 月至 2009 年 12 月

Ethics Committee/Institutional Review Board

Approval of Clinical Trial

Protocol Title：The Additional Therapeutic Effects of Virtual Reality System in Children with Developmental Delays
Protocol Version/Date：Version 1/27 October 2008
IRB Reference Number：97E-037
Chief Principal Investigator：Ru-Lan Hsieh
Informed Consent Form/Date：Version 1/27 October 2008
Investigator's Brochure/Date：NA
Board Meeting/Approval Dated：No.1/15-Jan-2009
Study Approval Expires：December 2009

The above study is approved by the Institutional Review Board on 1/15/2009.

在有效期屆滿之前，研究計畫主持人應向本委員會報告研究計畫的進行狀況。若屆時尚未完成，應重新申請。該計畫任何部分若欲更改，需向本委員會重新提出申請。計畫主持人對受試者任何保具有危險而且未能預期之問題，例如：對藥物、放射性元素或對醫療器材產生嚴重或非預期不良反應等，需立即向本委員會提出書面報告。

Yours Sincerely,
Gong-Jhe Wu, M.D.,Ph.D.
Chairman
EC/IRB,SKMH
Shin Kong Wu
Ho-Su Memorial Hospital
Taiwan R.O.C.

本院醫學倫理暨人體試驗委員會之組織與執行皆符合 ICH-GCP
The Ethics Committee/Institutional Review Board of Shin Kong Wu Ho-Su Memorial Hospital performs its functions according to written operating procedures and complies with GCP and with the applicable regulatory requirements.
審查案號 97E-037B
