# Supplementary material for: The Impact of Short-Term Video Games on Performance among Children with Developmental Delays: A Randomized Controlled Trial
Source: PLoS One. 2016 Mar 16;11(3):e0149714. doi: 10.1371/journal.pone.0149714 (PMC4794225; doi:10.1371/journal.pone.0149714)
Supplement: S3 Text — (DOC) [file pone.0149714.s004.doc]

**題目：健康復健互動軟體對發展遲緩兒童之復健加成療效及生活
品質研究**

**研究背景：**


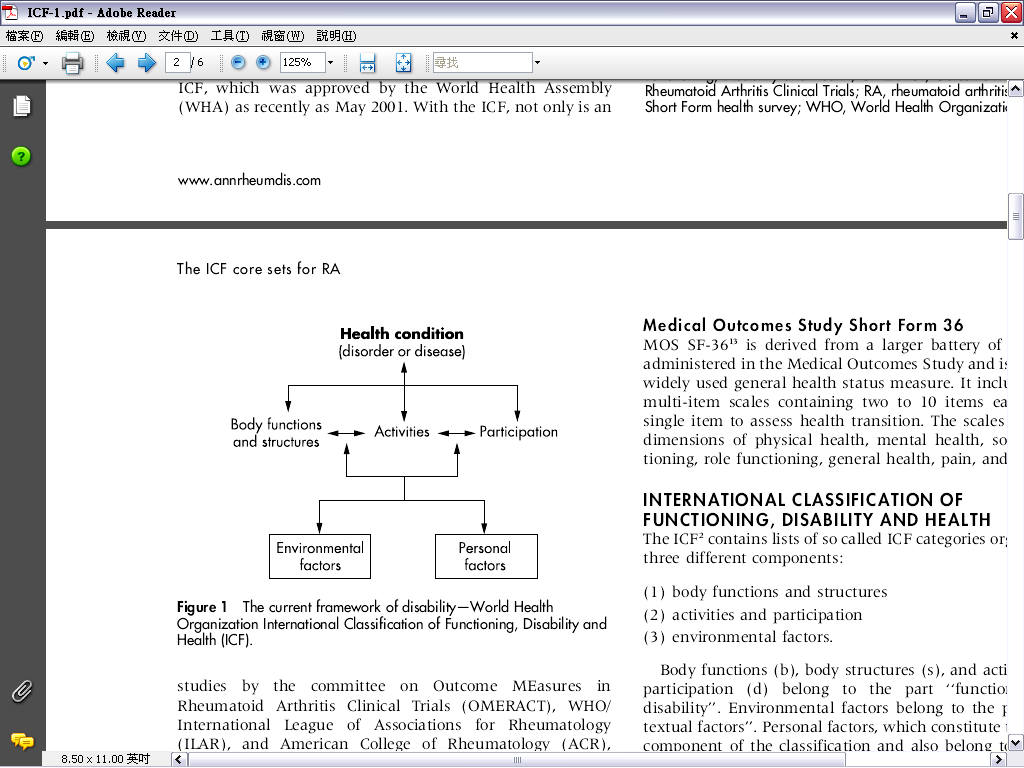
近年來，醫療照護由治療〝疾病為主〞的方式逐漸改為強調〝以病人為主〞的治療模式[1]。因此，2001年世界衛生組織提出將來的研究應以International Classification of Function, Disability, and Health (ICF) 為基礎，探討病患疾病嚴重度、生理功能、心理功能、功能性、運動性、情緒、社經地位、家庭支援，甚至包括照顧者之負擔、情緒等與治療療效及生活品質之相關性，進而促進病患之健康[2-6]，如圖一。

尤其針對發展中的兒童而言，其健康狀態更非以「疾病之有無」為判斷標準。以ICF之觀點而言，兒童健康係由發展、健康、社會、父母親及居住環境等多方面複雜的互動所影響[7]。據估計，約有10-15％的兒童因動作、行為或精神智能障礙等，需要特殊健康照護[8-10]。若這些兒童無法在早期獲得適當診斷與治療，將來長大後將無法在這充滿競爭性的社會中適應。

在台灣，全民健康保險制度於1995年開辦以來，目前的投保率已高達99％[11]。由1997年起，政府更推出兒童發展遲緩早期療育業務，國內疑似發展遲緩兒童皆可接受各醫療機構早期療育團隊之發展遲緩評估鑑定。鑑定成員包括復健科、心智科、眼科、耳鼻喉科、小兒科等專科醫師，職能、物理、語言治療師、臨床心理師、聽力師、社工師等，透過早期療育團隊的詳細問診、身體檢查及標準化工具，評估大動作、精細動作、認知、語言、社會及情緒等之發展情形，以鑑定兒童是否有發展遲緩。評估後若確定有發展遲緩，再針對發展遲緩部分接受早期療育，以提供兒童最大的復健療效[10, 12]。早期療育除強調改善發展各項次的功能及技巧外，更強調增進家長照護能力、促進兒童及家庭的健康，進而促進發展遲緩兒童能融合進入學校及社區[13]。以台北市為例，民國97年度之總評估人數為2,377人，較民國90年之1,012人高出2.3倍，總療育人次為100,412人次數，較民國90年之62,160人次增加1.6倍。由此可知，國內有這方面需求之兒童的確逐年在增加。

每位發展遲緩兒童之impairment皆會造成不同的disability。如腦膜炎、早產兒、腦室水腫等任何原因皆可能導致神經運動功能受損引起視力障礙（包括內外斜視，視力不良等），聽力障礙，口腔運動功能不良導致飲食及語言障礙，腸胃症狀如胃食道異常反射及便秘，張力、肌力不一導致肢體發展不均衡，大小便控制異常導致大小便失禁，智能障礙導致學習、認知受影響及抽搐等情形。功能上則包括坐起、站立、行走等之獨立性受到重大影響，異常張力及不足之肌力亦導致肌耐力不足及關節彎縮，進而影響移位、行走、處理日常生活自理時之平衡協調。因此，在探討發展遲緩兒童之復健療育時，應在ICF之觀念考量下，結合兒童健康狀況、身體結構、功能、環境因素、個人因素、參與程度、活動情形等各項因素再擬定復健療育治療計畫[2-6]。

在復健領域裡已有物理、職能、語言及心理復健等方法介入，而復健應用互動軟體主要是應用電視螢幕或投影設備及實物感應裝置，進行維持身體功能的體能及認知、協調、平衡訓練，目前僅有少數文獻探討關於類似虛擬實境等遊戲設備應用於中風、阿茲海默患者等研究報告，但關於發展遲緩兒童使用復健應用互動軟體是否可促進其發展等正式研究報告仍付之闕如，故本研究著重在客觀評估此進而討論其對發展遲緩兒童的復健療效及日常生活品質之長期影響。

**研究目的：**

評估使用健康復健互動軟體在發展遲緩兒童復健治療之加成療效。

**研究方法、進行步驟及執行進度：**

1. 收集本院發展遲緩兒童正在接受復健治療者。
2. 由研究人員依患童能力進行「Hot Plus 健康服務-復健互動遊戲」虛擬實境訓練，隨機分配A、B兩組，其中A治療方式為原本預訂的復健四週，四週後加上兩次每次30分鐘健康互動軟體，共四週；B組治療方式除了原本預訂的復健治療方式加上每週兩次，每次三十分鐘的健康復健互動軟體，四週後進行原本預訂的復健治療共四週。
3. 評估及治療方式：
4. 經復健科醫師詳細評估後，收集兩組至少各52位年紀為三歲以上發展遲緩的兒童，以可以聽懂簡單指令並父母願意接受配合接受健康復健互動治療為主。
5. 每位個案接受三次測量，訓練期為期四週，每週兩次，一次三十分鐘，每週治累計六十分鐘之治療時間，詳如下圖流程，如圖二。
6. 利用「Hot Plus 健康服務-復健互動遊戲」進行虛擬實境訓練計畫，於不同時期依個案進步情形逐漸引導及增加訓練遊戲難度。

**甲組**

治療前評估（T_0_）

四週 B：傳統復健治療

+

Hot Plus治療

**乙組**

治療前評估（T_0_）

四週評估（T_1_）

四週評估（T_1_）

八週評估（T_2_）

八週評估（T_2_）

四週 B：傳統復健治療

+

Hot Plus治療

四週 A：傳統復健治療

圖二‧比較甲、乙兩組，T_0_ – T_1_, T_1_ – T_2_, T_0_ – T_2_之差異，

除可明瞭Hot Plus是否有加成作用保留探討其療效是否

有持續性之效果。

四週 A：傳統復健治療

1. 每位受試者於治療前，治療後的第四週及第八週由研究人員進行以下列檢查評估，評估對象包括發展遲緩兒童及主要照護者。
   1. 發展遲緩兒童
2. Pediatric Quality of Life Inventory (Peds QL)
   共有21項題目，包括physical, emotional, social, 及school四個領域的功能，其分數愈高代表生活品質愈好。此外，亦可分為psychosocial health summary score, physical health summary score及total score等三個面相，分數為0至100分，愈高代表愈好。
3. 小兒健康評估問卷 (PODCI)
   評估兒童的一般健康，骨骼及肌肉狀態，共28項領域，其中包括physcal, psychological, social 及enviroment四個面向。
   1. 主要照護者
4. 情緒評估(Anxiety and fatigue)

Hospital Anxiety and Depression

1. Family Impact Module：共有36項題目，包括physical function, social, cognitive, communication, worry, daily activities及family relationships等八個領域之功能，分數愈高代表功能愈佳。
2. 健康照護滿意度
   Healthcare Satisfaction Generic Module （健康照顧滿意度）：可分成information, inclusion of family, technical skills, emotional need及overall satisfaction六項領域，其中分數愈高滿度愈好。此外並總滿意度之評分，其分數為0到100分。
3. 主要照護者生活品質WHO-QOL
   共28項領域，其中包括physcal, psychological, social 及enviroment四個面向。

**預期成果：**

1. 明瞭健康復健互動軟體對發展遲緩兒童復健之加成療效及生活品質之影響。
2. 明瞭健康復健互動軟體對主要照護者之情緒、衝擊及生活品質之影響。

**參考文獻**

- 1. Cushman LA, Stein K, Duffy CJ. Detecting navigational deficits in cognitive aging and Alzheimer disease using virtual reality. Neurology. 2008 Sep 16;71(12):888-95
  2. Broeren J, Claesson L, Goude D, Rydmark M, Sunnerhagen KS. Virtual rehabilitation in an activity centre for community-dwelling persons with stroke. The possibilities of 3-dimensional computer games. Cerebrovasc Dis. 2008;26(3):289-96.
  3. Piron L, Turolla A, Tonin P, Piccione F, Lain L, Dam M. Satisfaction with care in post-stroke patients undergoing a telerehabilitation programme at home. J Telemed Telecare. 2008;14(5):257-60.
  4. Lloyd J, Riley GA, Powell TE. Errorless learning of novel routes through a virtual town in people with acquired brain injury. Neuropsychol Rehabil. 2008 Jul 1:1.
  5. Buxbaum LJ, Palermo MA, Mastrogiovanni D, Read MS, Rosenberg-Pitonyak E, Rizzo AA, Coslett HB.Assessment of spatial attention and neglect with a virtual wheelchair navigation task. J Clin Exp Neuropsychol. 2008 Aug;30(6):650-60.
  6. Ohyama S, Nishiike S, Watanabe H, Matsuoka K, Takeda N. Effects of optokinetic stimulation induced by virtual reality on locomotion: a preliminary study. Acta Otolaryngol. 2008 Apr 2:1-4.
  7. Kang YJ, Ku J, Han K, Kim SI, Yu TW, Lee JH, Park CI. Development and clinical trial of virtual reality-based cognitive assessment in people with stroke: preliminary study. Cyberpsychol Behav. 2008 Jun;11(3):329-39.
  8. Cho S, Ku J, Park J, Han K, Lee H, Choi YK, Jung YC, Namkoong K, Kim JJ, Kim IY, Kim SI, Shen DF. Development and verification of an alcohol craving-induction tool using virtual reality: craving characteristics in social pressure situation. Cyberpsychol Behav. 2008 Jun;11(3):302-9.
  9. Broeren J, Bjorkdahl A, Claesson L, Goude D, Lundgren-Nilsson A, Samuelsson H, Blomstrand C, Sunnerhagen KS, Rydmark M. Virtual rehabilitation after stroke. Stud Health Technol Inform. 2008;136:77-82.
  10. Yancosek K, Daugherty SE, Cancio L. Treatment for the service member: a description of innovative interventions. J Hand Ther. 2008 Apr-Jun;21(2):189-94; quiz 195.
  11. Theodoros D, Russell T. Telerehabilitation: current perspectives. Stud Health Technol Inform. 2008;131:191-209.
  12. Koenig A, Wellner M, Köneke S, Meyer-Heim A, Lünenburger L, Riener R. Virtual gait training for children with cerebral palsy using the Lokomat gait orthosis. Stud Health Technol Inform. 2008;132:204-9.
  13. Goffredo M, Bernabucci I, Schmid M, Conforto S. A neural tracking and motor control approach to improve rehabilitation of upper limb movements.J Neuroeng Rehabil. 2008 Feb 5;5:5.
