# Supplementary material for: The Impact of Short-Term Video Games on Performance among Children with Developmental Delays: A Randomized Controlled Trial
Source: PLoS One. 2016 Mar 16;11(3):e0149714. doi: 10.1371/journal.pone.0149714 (PMC4794225; doi:10.1371/journal.pone.0149714)
Supplement: S4 Text — (DOC) [file pone.0149714.s005.doc]

Clinical Trial Protocol-English version

(Main points of the protocol of the Chinese version)

Investigator Signature：Ru-Lan Hsieh

| **Protocol title：**  The additional therapeutic effects of virtual reality system in children with developmental delays |
| --- |
| **Objectives：**  To identify the additional therapeutic effects of virtual reality system for children with developmental delays attending traditional rehabilitation programs |
| **Background：**  The International Classification of Functioning, Disability and Health (ICF) was developed by the World Health Organization (WHO) in 2001. The ICF contains 2 major parts, each consisting of 2 separate components. Part 1 is relevant to functioning and disability, and includes the components (1) *Body functions and structures* and (2) *Activities and participation*. Part 2 addresses contextual factors, and includes the components (1) *Personal factors* and (2) *Environmental factors*. A person’s functional health condition is viewed as a dynamic interaction between the ICF components. Under the ICF model, a child’s health can be affected by dynamic interaction among functional, behavioral, social, and environmental factors and health-related quality of life (HRQOL).  The prevalence of developmental delays in children is around 10% to 15%. If the developmental delays do not improve at school-age, children will face poor emotional, behavioral, and social interaction problems. Therefore, early diagnosis with intervention by family and early developmental expertise will improve the developmental delays in preschool children and decrease the risk for future leaning disability or poor social interaction.  Virtual reality system by interactive video game play has been popular in recent years. However, the effects of virtual reality system by interactive video game play on health-related quality of life and functional performance in children with developmental delays and their family impact remain undetermined. Therefore, a prospective study will be conducted to investigate the effects of short-term virtual reality system by using interactive-video-game playing on children with developmental delays attending traditional rehabilitation programs. |
| **Study Design：**  A prospective study. |
| **Methods：**  The participants will be randomly assigned to either Group A or Group B based on computer-generated random numbers. The traditional rehabilitation treatment comprised two stages of interactive-video-game playing. The Group A participants received traditional rehabilitation treatment alone for 4 weeks, followed by traditional rehabilitation treatment and additional eight 30-minutes sessions of interactive-video-game playing for 4 weeks.  The Group B participants received traditional rehabilitation treatment and additional eight 30-minutes sessions of interactive-video-game playing for 4 weeks, followed by traditional rehabilitation treatment alone for 4 weeks.  Following the recruitment and baseline assessment, the outcome measures will be assessed before treatment (Time 0), at the end of the first intervention in the fourth week (Time 1), and at the end of the second intervention in the eighth week (Time 2). The investigator conducting the assessment will be blinded to the allocation of each participant.  **Health-related quality of life and functional performance of the children**  Pediatric Quality of Life Inventory Generic Core Scales (PedsQL; parent proxy-report format): measure the pediatric HRQOL according to a parent proxy-report. The physical health summary scores, psychosocial health summary scores, and total scale scores will be calculated.  Parent-report form of the Pediatric Outcomes Data Collection Instrument (PODCI): assess the functional performance of the children. This study focus on the four functional performance domains in the PODCI: upper extremity and physical function, transfer and basic mobility, sports and physical functioning, and global functioning.  **Family impact**  The PedsQL Family Impact Module: assess family functioning.  The PedsQL Health Satisfaction questionnaire: assess the parents’ satisfaction with their child’s health care.  The World Health Organization Quality of Life Brief Version (WHOQOL-BREF) questionnaire: evaluate the parents’ QOL .  **Statistical analysis**  Chi-squared or *t* tests will be used to compare the differences in the data between Groups A and B according to their demographic and baseline variables. Between-group differences in the change from baseline in outcome measures will be conducted by paired *t* tests. All the statistical tests are two-tailed and α = 0.05. |
| **Effect：**   1. Investigate the effects of short-term virtual reality system by using interactive-video-game playing for children with developmental delays, including health related quality of life and functional performance. 2. Investigate the effects short-term virtual reality system by using interactive-video-game playing for family impact, including family functioning, parents’ satisfaction with their child’s heath, and quality of life. |
|  |
