# Supplementary material for: The Impact of Short-Term Video Games on Performance among Children with Developmental Delays: A Randomized Controlled Trial
Source: PLoS One. 2016 Mar 16;11(3):e0149714. doi: 10.1371/journal.pone.0149714 (PMC4794225; doi:10.1371/journal.pone.0149714)
Supplement: S5 Text — (PDF) [file pone.0149714.s006.pdf]

Protocol Registration Receipt
07/08/2014

Effects of Virtual Reality System in Children With Developmental Delays

This study has been completed.

Sponsor:	Ru-Lan Hsieh
Collaborators:	
Information provided by (Responsible Party):	Ru-Lan Hsieh, Taipei Medical University
ClinicalTrials.gov Identifier:	NCT02184715

Purpose

The purpose of this study is to investigate the effects of virtual reality system on children with developmental delays.

Condition	Intervention	Phase
Developmental Delays	virtual reality system	Phase 4

Study Type: Interventional
Study Design: Health Services Research, Crossover Assignment, Single Blind (Outcomes Assessor), Randomized, Efficacy Study
Official Title: The Additional Therapeutic Effects of Virtual Reality System in Children With Developmental Delays

Further study details as provided by Ru-Lan Hsieh, Taipei Medical University:

Primary Outcome Measure:

- health of children (Pediatric Quality of Life Inventory)   [Time Frame: changes from baseline at one and two months.] [Designated as safety issue: No]

  Following the recruitment and baseline assessment, outcome measures were assessed before treatment (Time 0), at the end of first intervention in the fourth week (Time 1), and at the end of second intervention

in the eighth week (Time 2).

Enrollment: 157
Study Start Date: January 2009
Study Completion Date: December 2011
Primary Completion Date: January 2011

Arms	Assigned Interventions
Active Comparator: Virtual reality video game<br>Participants received rehabilitation treatment and additional virtual reality system (30 minutes of interactive virtual reality system play, two times per week, in eight sessions over a 4-week span) for 1 month, followed by rehabilitation treatment for 1 month	virtual reality system<br>The participants were randomly assigned to either Group A or Group B. Group A received rehabilitation treatment and additional virtual reality system for 1 month, followed by rehabilitation treatment for 1 month; by contrast, the participants in Group B received rehabilitation treatment for 1 month, followed by rehabilitation treatment and additional virtual reality system for 1 month.
Placebo Comparator: Virtual reality system<br>received rehabilitation treatment for 1 month, followed by rehabilitation treatment and additional virtual reality system (30 minutes of interactive video game play, two times per week, in eight sessions over a 4-week span) during the one month of intervention period.	virtual reality system<br>The participants were randomly assigned to either Group A or Group B. Group A received rehabilitation treatment and additional virtual reality system for 1 month, followed by rehabilitation treatment for 1 month; by contrast, the participants in Group B received rehabilitation treatment for 1 month, followed by rehabilitation treatment and additional virtual reality system for 1 month.

Participants attended eight 30-minute sessions of virtual reality system for 4 weeks in addition to regular rehabilitation programs.

Eligibility

Ages Eligible for Study: 2 Years to 12 Years
Genders Eligible for Study: Both

Inclusion Criteria:

- confirmed to have developmental delays
- provided informed consent
- 2 to 12 years old

Exclusion Criteria:

- failed to provide informed consent

Contacts and Locations

Locations

Taiwan

Shin Kong Wu Ho-Su Memorial Hospital
Taipei, Taiwan, 111-01

Investigators

Principal Investigator:	Ru-Lan Hsieh, MD	Shin Kong Wu Ho-Su Memorial Hospital; Taipei Medical University

More Information

Responsible Party:	Ru-Lan Hsieh, MD, Taipei Medical University
Study ID Numbers:	HP-01
Health Authority:	Taiwan: Department of Health
